# ABCDE approach to victims by lifeguards: How do they manage a critical patient? A cross sectional simulation study

**DOI:** 10.1371/journal.pone.0212080

**Published:** 2019-04-30

**Authors:** Felipe Fernández-Méndez, Martín Otero-Agra, Cristian Abelairas-Gómez, Nieves María Sáez-Gallego, Antonio Rodríguez-Núñez, Roberto Barcala-Furelos

**Affiliations:** 1 CLINURSID Research Group, University of Santiago de Compostela, Santiago de Compostela, Spain; 2 University College of Nursing, University of Vigo, Pontevedra, Spain; 3 Faculty of Education and Sport Sciences, REMOSS Network Research, University of Vigo, Pontevedra, Spain; 4 Faculty of Education Sciences, Universidade de Santiago de Compostela, Santiago de Compostela, Spain; 5 Institute of Health Research of Santiago (IDIS), Santiago de Compostela, Spain; 6 Faculty of Education, University of Castilla la Mancha, Toledo, Spain; 7 Pediatric Area, Pediatric Emergency and Critical Care Division, University Hospital of Santiago de Compostela, Santiago de Compostela, Spain; 8 Mother-Child Health and Development Network (Red SAMID), Carlos III Health Institute, Madrid, Spain; University of Palermo, ITALY

## Abstract

**Introduction:**

Decision-making in emergencies is a multifactorial process based on the rescuer, patient, setting and resources. The eye-tracking system is a proven method for assessing decision-making processes that have been used in different fields of science. Our aim was to evaluate the lifeguards’ capacity to perform the ABCDE (Airway-Breathing-Circulation-Disability-Exposure) approach when facing a simulated critically ill-drowned victim.

**Methods:**

A cross-sectional simulation study was designed to assess the skills and sequence of the ABCDE approach by 20 professional lifeguards. They had to assess a victim and act according to his/her clinical status by following the ABCDE primary assessment approach. The two kinds of variables were recorder: those related to the quality of each step of the ABCDE approach and the visual behaviour using a portable eye-movement system. The eye-tracking system was the Mobile Eye system (Bedford, USA).

**Results:**

None of the study participants were able to complete correctly the ABCDE approach. Lifeguards spent more time in the Circulation step: Airway (15.5±11.1 s), Breathing (25.1±21.1 s), Circulation (44.6±29.5 s), Disability (38.5±0.7 s). Participants spent more time in viewpoints considered as important (65.5±17.4 s) compared with secondary ones (34.6±17.4 s, p = 0.008). This was also represented in the percentage of visual fixations (fixations in important viewpoints: 63.36±15.06; fixation in secondary viewpoints: 36.64±15.06; p = 0.008).

**Conclusion:**

Professional lifeguards failed to fully perform the ABCDE sequence. Evaluation by experts with the help of eye-tracking technology detected the lifeguards’ limitations in the assessment and treatment of an eventual critically ill victim. Such deficits should be considered in the design and implementation of lifeguards’ training programmes.

## Introduction

Drowning is a public health problem identified by the WHO as one of the main causes of mortality and morbidity [1], and lifeguards are the professionals with the duty to intervene in an aquatic incident. In addition to environmental water risks, the current occupation and use of aquatic environments for leisure are very popular [2], so different medical complications (i.e., cardiac arrest, marine stings, injuries…) may also require the attention of lifeguards.

The actions that lifeguards perform when incidents happen in aquatic environments are defined by the drowning timeline which includes preparation, prevention, rescue and mitigation [3]. Mitigation refers to the skills for the evaluation and treatment of the victim after an incident.

Mitigation of the aquatic incident requires identifying the problem, establishing a preliminary diagnosis and making the appropriate decisions to treat drowning in a hostile environment [4]. Moreover, for each drowned person who dies, it has been estimated that four people receive care in the emergency services for non-fatal drowning [5].

Usually, when talking about drowning, collective thinking associates it with the worst scenario, that is the cardiorespiratory arrest for which the European Resuscitation Council (ERC) Guidelines 2015 recommend performing cardiopulmonary resuscitation (CPR) [6]. However, non-fatal drowning that does not necessarily need CPR but rather alternative urgent attention is much more prevalent [7]. And it is the one that requires an ABCDE approach to assess the signs and symptoms and to offer adequate immediate treatment [8].

Unlike other health professionals, for lifeguards, 99% of the actions are focused on prevention and rescue, and only 1% belong to medical assistance [7]. However, lifeguards have to be prepared for when this 1% of critical interventions occur, in which decision-making is essential. Decision-making in emergencies is a multifactorial process based on the rescuer, the patient, the setting and the resources, which is difficult to assess since it is an internal process that occurs rapidly [9].

To try to understand this decision-making process, the eye-tracking system was reported as a valid and reliable instrument. It is a proven method that has been used in different fields as sports sciences [10,11] or emergencies [12]. In addition, it is considered a great tool that might positively contribute to improving the lifeguards’ skills [13].

Therefore, the aim of the study was to systematically evaluate the decision-making and the capacity in the use of the ABCDE approach by lifeguards.

## Methods

### Sample

A convenience sample of 20 professional lifeguards [qualified in aquatic facilities (i.e., swimming pool…) and natural water environments (i.e., seaside…)] was invited to participate in this study. Participation was voluntary and authorized through written informed consent. All lifeguards were active professionals trained at the University of Vigo (Spain). Their rescue training was in accordance with regional laws (40 hours of first aid). The first aid training followed the recommendations of the ERC in terms of CPR [14] and prehospital trauma life support to train the ABCDE approach [15].

### Study design

A cross-sectional simulation study was designed to evaluate the skills and sequence of the ABCDE approach. This study was approved by the Ethics Committee of the Faculty of Education and Sports Sciences (University of Vigo—Spain) with code 04-1812-17.

### Simulating scenario

The simulating scenario was designed by a multidisciplinary group of experts (emergency doctors, nurses and first-aid coordinators), all of them simulation specialists and instructors. The manikin simulator was programmed according to the values in Table 1.

**Table 1:** Simulator programming.

**Airway**	**Airway obstruction by tongue fall due to decreased level of consciousness.**
**Breathing**	Respiratory rate of 40 breaths per minute. Superficial breaths.
**Circulation**	Heart rate of 140 beats per minute. Weak radial pulse and strong carotid pulse. Blood pressure of 90/60 mmHg.
**Disability**	The simulator did not respond to verbal stimuli. Facing painful stimuli, the patient responded with a scream.
**Exposure**	Wound due to erosion in left lower limb with small haemorrhage (distracting wound).

The participants received the indication that they would enter a room in which they should assess a victim and that they should act according to his/her clinical status following the ABCDE primary assessment approach. The room simulated a beach aid station. The usual material to attend the victim in a real case was available. The simulator remained in the same situation, unchanged, for 10 minutes at which time it went into PCR S1 Video). If the participants finished the ABCDE approach before 10 minutes, the instructor would configure the simulator into cardiac arrest (CA) immediately.

The subjective evaluation of the lifeguards’ performance was carried out by a BLS instructor [14] and two ALS [8,16]. To do this, a checklist was used to evaluate the ABCDE primary assessment based on the recommendations of the Prehospital Trauma Life Support [15] (S1 Checklist).

### Variables

Demographic data such as sex, age, height, weight and body mass index (BMI) were recorded. Afterwards, variables related to the training of the participants were recorded through a questionnaire: last training, knowledge of ABCDE approach, if she/he had ever had to perform the ABCDE approach, knowledge of AED and if she/he had done any simulation practice.

Variables regarding the ABCDE approach are shown in Table 2. Eye-tracking allowed the collection of data of views from the located point and percentage of time in which the located point was viewed from the following areas (defined as viewpoints of great importance): Airway (mouth), Breathing (neck, thorax, abdomen), Circulation (arm, leg, hand, haemorrhage, carotid pulse, radial pulse), Disability (eyes) and Exposure (thermal blanket) and AED. The rest of the viewpoints were classified as unimportant.

**Table 2:** Capacity to perform the ABCDE approach.

ABCDE steps	ASSESSED?	SUB-STEPS ASSESSED	CRITERIA
**Airway**	Assess airway	Assess the airway correctly.	Observe the inside of the mouth for foreign bodies and/or secretion and perform manoeuvre to open the airway or use an oropharyngeal cannula.
		Correct order in which the airway is assessed.	
**Breathing**	Assess breathing	Assess the breathing correctly.	Observe if the patient breathes, chest symmetry and presence of wounds in the thorax.
		Correct order in which the breathing is assessed.	
		Check if the patient is breathing.	
		Check the thoracic symmetry.	
**Circulation**	Assess circulation	Assess the circulation correctly.	Observe the presence of haemorrhage, peripheral and central pulses.
		Correct order in which the circulation is assessed.	
		Check central pulse.	
		Check peripheral pulse.	
		Check the quality of the pulse.	
		Check the presence of haemorrhage.	
**Disability**	Assess disability	Assess the neurological status correctly.	Evaluate according to the AVPU scale.
		Correct order in which the neurological status is assessed.	
**Exposure**	Asses exposure	Assess exposure correctly.	
**Cardiac arrest recognition**		Recognise cardiac arrest.	
**Perform CPR with AED**		Use AED.	

### Instruments

The evaluation was performed on a manikin ALS Simulator (Laerdal, Stavanger, Norway). The gaze fixation variables were obtained through the Mobile Eye gaze tracking system of the ASL laboratories (Bedford, USA). It is based on lightweight glasses that support two cameras: one of them records the scene and the other records the point where the vision is focused (extracted by the reflection produced by the cornea and the pupil in a lens). Both signals are registered through its DVCR recording unit and integrated into one via the computer system. This gives us a joint view of the environment observed by the participant and the visual fixations performed. The Mobile Eye system was calibrated using the Eye Vision 2.2.5 software. The resulting videos (supplementary file) have been analysed using the ASL Result Plus Gaze Map software. Both installed in the ACER ASPIRE 5920G laptop (Make INC, Taipei, Taiwan).

### Statistical analysis

The statistical analysis was performed with the Windows statistical package IBM SPSS Statistics version 20 (SPSS, Chicago, Illinois, USA). Continuous variables were described according to measures of central tendency (mean) and dispersion (standard deviation). Categorical variables were described according to absolute and relative frequencies. To verify the normality of the sample, the Kolmogorov-Smirnov test was performed. The Wilcoxon Signed Rank Test was used to verify the differences between the viewpoints of great importance in the ABCDE approach and the unimportant ones, and also to find differences between the percentage of fixations and the percentage of fixation time at each point (significance level of p < 0.05 in both analyses).

## Results

### Characteristics of participants

A total of 18 men and 2 women participated in the study aged between 28.35±6.90 years old, weight of 73.30±8.39 kg, height of 176.40±6.58 cm and BMI of 23.51 ± 2.04 kg·m^-2^.

The last formal training of the participants was 31.40±35.40 months before the study. Half of the participants received their last accredited training one year prior to the study. All participants knew what the ABCDE approach was, although 55% had not used it nor had previously performed a simulation scenario test.

### ABCDE approach

**Fig 1:**
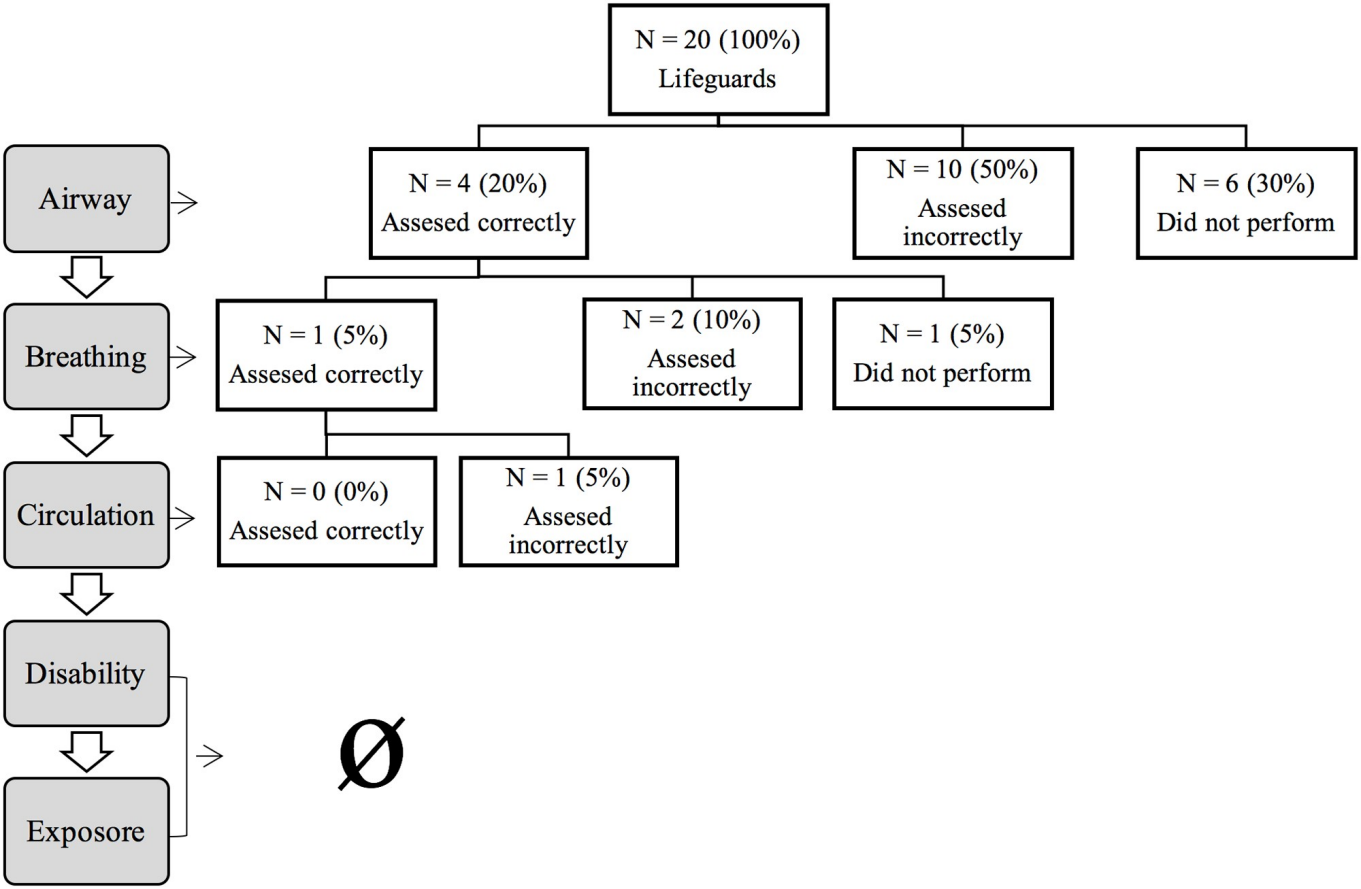
Descriptive analysis about participants’ capacity to perform ABCDE approach.

None of the study participants managed to complete the primary assessment correctly (Fig 1). Regarding the results of the assessment parts:

*Airway*: 45% of the participants performed the airway approach as the first step. 30% of the participants did not take this step and half did it incorrectly.

*Breathing*: This part of the primary assessment was carried out by 55% of the participants in second place, and 15% did not contemplate it. 25% of the participants correctly assessed breathing. 80% checked the victim’s breathing, and 30% checked the symmetries of the victim’s thorax.

*Circulation*: 60% of the participants assessed the circulation, but none did it correctly and less than half did it in third place (40%). The majority of the participants did not check pulses, and 35% did not perform the assessment of potential haemorrhages.

*Disability*: Only two participants evaluated this step, one in second and another in fourth place.

*Exposure*: 25% of the participants performed this step.

*CA recognition and performance*: Half of the participants knew how to recognize the CA at the time it took place, but only 35% of the participants used an AED together with CPR.

### Fixations during ABCDE approach (eye-tracking)

Data from four lifeguards has been lost due to technical issues. No significant differences were observed in any of the vision points when comparing the percentage of fixations and the percentage of fixation time (p > 0.05).

Tables 3 and 4 show the total percentage of the fixings of the most important viewpoints compared with the unimportant. In addition, the percentage of fixings of each important viewpoint with respect to the total. Regarding time, the time spent in each viewpoint is expressed in percentage, and the time spent in each part of the ABCDE approach is shown in seconds.

**Table 3:** Descriptive analysis of fixings performed by participants during the ABCDE approach (total, airway and breathing).

N = 16 (4 lost)		Mean (SD)	CI	Wilcoxon Signed Rank Test
**Viewpoints of great importance**	**Fixings** (%)	63.36 (15.06)	55.34–71.39	Total significantly point-views *vs*. Total insignificantly point-views **(p = 0.008)**
	**Time** (%)	65.45 (17.36)	56.20–74.70	
**Unimportant viewpoint**	**Fixings** (%)	36.64 (15.06)	28.61–44.66	
	**Time** (%)	34.55 (17.36)	25.30–43.80	
**Airway**				
***Mouth***	**Fixings** (%)	17.58 (9.78)	12.37–22.79	
	**Time** (%)	18.16 (11.11)	12.24–24.08	
***Time in Airway*** (s)		15.50 (9.17)	10.20–20.80	
**Breathing**				
***Neck***	**Fixings** (%)	4.03 (3.43)	2.20–5.86	
	**Time** (%)	4.87 (5.43)	1.98–7.76	
***Abdomen***	**Fixings** (%)	5.81 (3.32)	4.03–7.58	
	**Time** (%)	7.72 (7.93)	3.50–11.95	
***Thorax***	**Fixings** (%)	9.73 (6.98)	6.01–13.44	
	**Time** (%)	9.80 (8.47)	5.29–14.32	
***Time in Breathing*** (s)		25.12 (21.12)	14.26–35.98	

Fixings and time of total and each viewpoint in percentage.

Time of each total section of the ABCDE approach in seconds.

**Table 4:** Descriptive analysis about fixings performed by lifeguards during the ABCDE approach (circulation, disability, exposure and AED).

N = 16 (4 lost)		Mean (SD)	CI
**Circulation**			
***Hand***	**Fixings** (%)	1.40 (1.79)	0.45–2.35
	**Time** (%)	1.93 (2.72)	0.48–3.38
***Arm***	**Fixings** (%)	6.69 (5.76)	3.61–9.76
	**Time** (%)	5.98 (7.40)	2.03–9.92
***Leg***	**Fixings** (%)	4.06 (5.63)	1.06–7.06
	**Time** (%)	3.65 (5.90)	0.51–6.79
***Haemorrhage***	**Fixings** (%)	6.99 (6.96)	3.28–10.70
	**Time** (%)	6.51 (6.01)	3.30–9.71
***Carotid pulse***	**Fixings** (%)	0.26 (0.84)	-0.18–0.71
	**Time** (%)	0.32 (1.04)	-0.23–0.86
***Radial pulse***	**Fixings** (%)	0.15 (0.44)	-0.08–0.38
	**Time** (%)	0.19 (0.57)	-0.11–0.49
***Time in circulation*** (s)		44.58 (29.45)	25.87–63.30
**Disability**			
***Eyes***	**Fixings** (%)	0.93 (1.27)	0.25–1.60
	**Time** (%)	1.21 (1.81)	0.25–2.18
***Time in Disability*** (s)		38.50 (0.71)	32.15–44.85
**Exposure**			
***Thermal blanket***	**Fixings** (%)	4.15 (8.27)	-0.26–8.56
	**Time** (%)	3.75 (7.22)	-0.10–7.60
**AED**			
**Fixings** (%)		1.60 (2.46)	0.29–2.91
**Time** (%)		1.35 (2.19)	0.18–2.51

Fixings and time of total and each viewpoint in percentage.

Time of each total section of the ABCDE approach in seconds.

**Fig 2:**
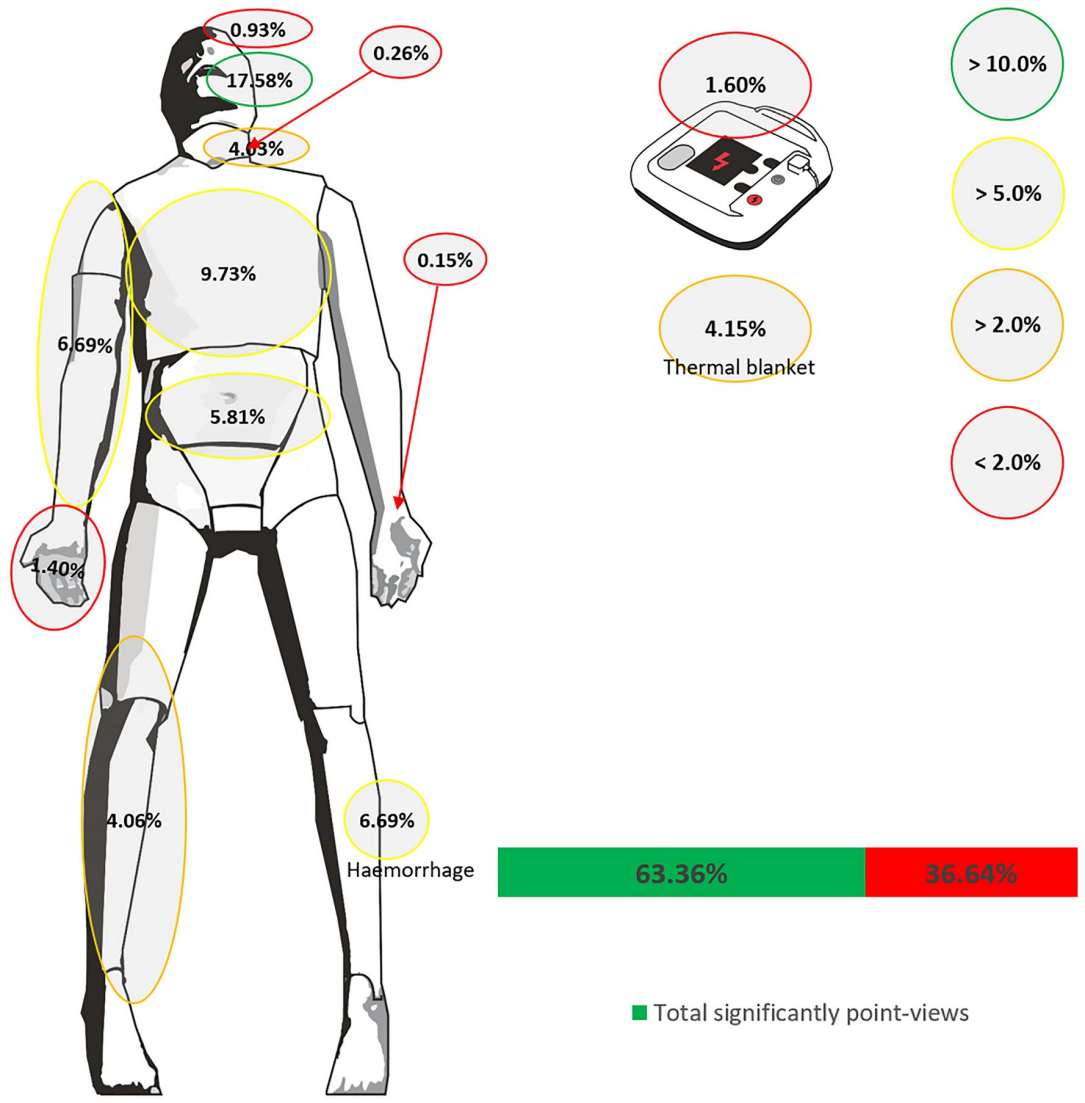
Descriptive analysis about fixings performed by participants during ABCDE approach.

Around 60% of the fixations and fixation time were devoted to important areas of vision for the ABCDE approach (Fig 2 and Table 3). Significant differences were found compared with the percentage of fixations and fixation time of unimportant areas (p < 0.008 in both cases).

The "Circulation" approach was the one that required more time to carry out its assessment (44.6 s), followed by "Disability" (38.5 s) and "Breathing" (25.1 s). The steps that required less time were "Exposure" (3.8 s) and "Airway" (15.5s). The fixings and time of each step of the ABCDE approach are shown in Tables 3 and 4.

## Discussion

Simulation training is a well-recognised educational tool in medical and emergency personnel education that allows the improvement of technical and non-technical skills [17–19]. This type of training is not only very useful in medical training for emergency professionals, but also for bystanders [20–22]. Eye-tracking technology was reported as an effective instrument to complement the simulation training and the subjects performance assessment [23,24]. In addition, it is an evaluation tool that complements the information reported by experts and thus the effectiveness of the training. Recent publications show its effectiveness in assessing or evaluating in simulating scenarios related to anaphylaxis or paediatric trauma [12,25]. In fact, the accuracy of this tool also resulted in it being used in real situations [26].

In our study, eye-tracking was used to analyse the lifeguards’ ABCDE approach skills. Professional lifeguards showed differences when it came to recognize and treat a critically ill patient in a simulated scenario. Although all the participants had theoretical training and knew what was and how the primary assessment was applied; they had not received simulation training, and half of them had not used the primary assessment in a real situation. Although there is no clear evidence that training "life support in trauma" has an impact on the results of trauma victims, there is evidence that educational initiatives improve knowledge about what to do in emergency situations [27].

Most rescuers of this study evaluated the Airway (14 of 20: 70%) and Breathing (17 of 20: 85%) although only 4 (20%) and 5 (25%) assessed both correctly. Only 40% evaluated Circulation. Of the subjects who performed the evaluation of Circulation, only 3 took a central pulse, and 2 checked a peripheral pulse. The assessment of Circulation was the step that required the longest assessment time (45 s). Maybe this was due to the haemorrhage that the simulator presented in the lower limb as a distracting factor introduced by the researchers. The haemorrhage was small and produced by an erosion. The majority of the participants did not consider assessing pulses neither peripheral nor central. This could be due to the fact that the Guidelines of the ERC as well as the Guidelines of the American Heart Association of basic life support stress that taking the pulse is not a necessary measure to establish the diagnosis of cardiorespiratory arrest [14,28]. However, the simulator did not present a PCR and required a Circulation evaluation.

The application of a structured assessment system has become the norm in trauma. This approach to the early recognition and treatment of life-threatening injuries has been trained in trauma courses for decades [29,30]. In the study by Olgers et al. [31] in which they investigated the use of the ABCDE approach by emergency doctors, they observed that this approach was used in 26% of patients. When the ABCDE approach was used, it was performed in the correct way by the majority of the sample (83%). The reason why the doctors decided not to use this assessment approach was because of the general clinical impression, the vital signs registered by nursing or that the reason for the consultation did not suggest an unstable patient [31]. In another study conducted in a hospital emergency department, it was found that only 52% of patients were evaluated with the ABCDE approach, and 17% were fully evaluated with precision [32].

In view of our results, it seems that the lifeguards are not competent in performing the ABCDE approach in a simulated scenario. With respect to the visual fixations, the lifeguards were able to maintain adequate attention and fixation, which means that they are focused on looking. Around 60% of the fixations and the time of visual fixations were dedicated to important areas of vision for the ABCDE approach. This is independent of the decisions made at each moment because the eyes could be focusing on the important parts and not knowing the decisions to make.

### Practical implications

The poor results found in this study are relevant and useful to re-design and to re-organize the training of lifeguards in two ways: the initial training and re-training over time.

More efforts are needed to train in terms of the ABCDE approach, considering the correct order of the different steps and its relevance in the sequence. As well as training of other competencies as CPR, the use of feedback devices is strongly recommended, and the implementation of useful and new training and evaluation tools should be implemented to teach the ABCDE approach. Nevertheless, the changes in lifeguards’ training should not only be made in terms of technology, using feedback devices or eye-tracking, which could involve an economic investment that might not be affordable for every institution. The better the capacity of the teacher, the better the training. Therefore, instructors should have better skills to teach, and have role-playing debriefing skills, an important role in simulation-based education. Good debriefing skills allows more reflexive practice, helping the lifeguards to identify their own errors and performance gaps [33]. In addition, the ABCDE approach comprises a set of different skills and knowledge whose competency might decrease over time. Hence, lifeguards’ training should consider this aspect and periodic re-training should be mandatory to maintain the skills quality.

### Limitations

This is a simulation study; therefore, a real intervention could generate different results, which we suppose might be even worse than those observed in our trial. The lack of experience of the participants in the use of the simulation methodology could be a limiting factor. The subjects’ sample was small, so the results should be taken with caution. Also, the duration of the scenario could be a limiting factor as well because we do not know what might happen if the scenario had lasted longer. The participants knew that they were under observation, and this may have modified their performance.

## Conclusions

Professional lifeguards showed limited practical skills in a simulated scenario that required the ABCDE approach, which was evaluated by experts and eye-tracking technology. Such deficits should be considered in the re-design and implementation of lifeguards’ training programmes.

## Supporting information

S1 Video Video recording of eye-tracking system. (MP4)

S1 Checklist Evaluation tool of the lifeguards’ performance. (DOCX)
