# Supplementary material for: ABCDE approach to victims by lifeguards: How do they manage a critical patient? A cross sectional simulation study
Source: PLoS One. 2019 Apr 30;14(4):e0212080. doi: 10.1371/journal.pone.0212080 (PMC6490899; doi:10.1371/journal.pone.0212080)
Supplement: S1 Checklist — (DOCX) [file pone.0212080.s002.docx]

| CODE |  | AGE |  |
| --- | --- | --- | --- |
| MAN | WOMAN | HEIGHT |  |
| Date of last training in first aid | | |  |

1. Do know what primary assessment is? YES NO
2. Have you ever had to perform a primary assessment? YES NO
3. Do you know how to do CPR under the current recommendations? YES NO
4. Have you done any real CPR? YES NO
5. Do you know how to use an AED? YES NO
6. Have you done any clinical simulation practice? YES NO

| A | Time to evaluate: | | | Correct  Correct order | | Incorrect  Incorrect order | |
| --- | --- | --- | --- | --- | --- | --- | --- |
| B | Time to evaluate: | | | Correct  Correct order | | Incorrect  Incorrect order | |
|  | Breathing assessment | | | Yes | | No | |
|  | Evaluation of thoracic symmetry | | | Yes | | No | |
| C | Time to evaluate: | | | Correct  Correct order | | Incorrect  Incorrect order | |
|  | Central pulse | | | Yes | | No | |
|  | Peripheral pulse | | | Yes | | No | |
|  | Looks for haemorrhages | | | Yes | | No | |
| D | Time to evaluate: | | | Correct  Correct order | | Incorrect  Incorrect order | |
|  | Check | Alert | Verbal | | Pain | | Null |
| E | Time to evaluate: | | | Correct | | Incorrect | |
|  | Prevention of hypothermia | | | Yes | | No | |
| CPR | CPR detection time | | | TIME | |  | |
|  | Use the AED | | | Yes | | No | |
| Comments: | | | | | | | |
